# Quality indicators and excellence requirements for a multidisciplinary lung cancer tumor board by the Spanish Lung Cancer Group

**DOI:** 10.1007/s12094-021-02712-8

**Published:** 2021-10-19

**Authors:** M. Guirado, A. Sanchez-Hernandez, L. Pijuan, C. Teixido, A. Gómez-Caamaño, Á. Cilleruelo-Ramos

**Affiliations:** 1 Medical Oncology Department, Hospital General Universitario de Elche, 03203 Elche, Spain; 2 Medical Oncology Department, Consorcio Hospitalario Provincial de Castellón, 12002 Castellón de la Plana, Spain; 3 Pathology Department, Bellvitge University Hospital, 08907 L’Hospitalet de Llobregat, Spain; 4 Thoracic Oncology Unit, Department of Pathology, IDIBAPS, Hospital Clinic of Barcelona, C. de Villarroel, 170, 08036 Barcelona, Spain; 5 Department of Radiation Oncology, Hospital Clínico Universitario Santiago de Compostela, 15706 Santiago de Compostela, Spain; 6 Thoracic Surgery Department, Hospital Clínico Universitario Valladolid, 47005 Valladolid, Spain

**Keywords:** Lung neoplasms (MeSH), Quality indicators (MeSH), Quality of health care (MeSH), Multidisciplinary team, Tumor board

## Abstract

Multidisciplinary care is needed to decide the best therapeutic approach and to provide optimal care to patients with lung cancer (LC). Multidisciplinary teams (MDTs) are optimal strategies for the management of patients with LC and have been associated with better outcomes, such as an increase in quality of life and survival. The Spanish Lung Cancer Group has promoted this review about the current situation of the existing national LC-MDTs, which also offers a set of excellence requirements and quality indicators to achieve the best care in any patient with LC. Time and sufficient resources; leadership; administrative and institutional support; and recording of activity are key factors for the success of LC-MDTs. A set of excellence requirements in terms of staff, resources and organization of the LC-MDT have been proposed. At last, a list of quality indicators has been agreed to achieve and measure the performance of current LC-MDTs.

## Introduction

Lung cancer (LC) is one of the most complex of all common cancers, given its heterogeneity, the evolution of the treatment options and the huge societal impact derived [1]. Different factors, such as the stage of the disease or histology, as well as patient’s age, comorbidities, symptoms, performance status and preferences, have an impact on its management. Active treatment options include surgery, radiotherapy and systemic therapy, the latter being frequently used as combinations of two or three molecules [2]. For all these reasons, tight coordination among multiple specialties is needed to decide the best therapeutic approach and to provide optimal care to LC patients.

Decision making through multidisciplinary teams (MDTs) is considered an essential and optimal strategy for the management of patients with cancer, since they position the patient at the center of the process. MDTs are alliances of medical and healthcare professionals involved in a specific tumor disease who, for each case, agree on evidence-based decisions and coordinate the delivery of care at all stages of the process [3–5]. Their main objective is to optimize health outcomes and improve patients’ care [5]. Effective MDT-driven care depends on the capacity of teamwork, the availability of information from patients, the presence of leadership, the optimal team and meeting management, and workload [6]. Literature addressing the duties of MDTs is diverse and growing [6]. The inclusion of MDTs has been associated with positive consequences in multiple dimensions of patients’ management, and with an increase in survival [7]. MDTs also facilitate the access to clinical trials and real world data [8, 9].

Particularly, MDTs for patients with LC (LC-MDTs) have been associated to an improvement in coordination and a reduction in the variability of patient care which, in turn, leads to a better patient experience. In addition, LC-MDTs facilitate the overcoming of barriers to treatment, promote standardized treatment, and allow the auditing of clinical services [10]. Multidisciplinary care of LC improves many clinical outcomes, among them, the survival of patients [2, 11, 12]; likewise, it increases adherence to treatment guidelines and the possibilities of receiving active treatment or any therapeutic option [2]. In the specific case of advanced LC, a reduction in the interval between diagnosis and treatment, and an increase in patients’ quality of life (QoL) have also been observed [13].

The progressive increase of the complexity of LC diagnosis and treatment requires a greater number and degree of specialization among the professionals involved in LC-MDTs [1, 3]. LC-MDTs are well accepted and have an inherent opportunity cost, and they need to be efficient, effective and flexible to be able to incorporate innovations and perform the best practice [14]. In this line, the measurement of the results in quality of care is essential to demonstrate that the interventions of the MDTs are cost-effective.

During times of the COVID-19 pandemic, the proper functioning of LC-MDTs is even more relevant to ensure the continuity of the presentation of cases. In this sense, a successful experience of transition from in-person to virtual thoracic tumor board has been presented as a feasible and efficient model for multidisciplinary management in the context of social distancing [15]. This model can also help to overcome previous distance barriers presented in many hospital centers.

The Spanish Lung Cancer Group (*Grupo Español de Cáncer de Pulmón*) is a cooperative group of oncologists that promote clinical research and multidisciplinary work in the field of LC. With this review and statement publication, the group aimed to: (a) review the current situation of the existing national LC-MDTs; (b) propose a practical, effective and efficient model, agile enough to be implemented in most Spanish hospitals, independently of their technologic and care capacity; and (c) offer a set of excellence requirements and quality indicators, to achieve the best care in any patient with LC, regardless of the geographic situation.

## Current situation of LC-MDTs

In general, clinicians agree that MDTs can improve care of patients with LC. However, some barriers are currently present at different levels, and they may hinder the successful implementation of a LC-MDT.

LC-MDTs require a great commitment in terms of the time dedicated to multidisciplinary care, and can jeopardize the optimization of the care work of health professionals. A Spanish qualitative study found that MDT members did not feel the recognition of their dedication as “real work” [16]. Time, although is often insufficient, is crucial for the preparation of the MDT meetings. Scarce time can complicate the preparation of the cases to present, or arise as a lack of information about the patient to properly discuss the case [17, 18]. This can end in inappropriate management plans, delays in the treatment onset, repeated discussions, as well as demotivation and stress in MDT members [19].

In the past, physicians were self-sufficient and considered themselves capable of managing patients on their own, but nowadays work within the MDTs seems necessary [18]. Differences of opinion about the treatment of a patient are natural and common, although sometimes can negatively affect interpersonal relationships and the functioning of the LC-MDT. Sometimes, there is also concern about a disagreement within the LC-MDT members and the lack of possibility to consulting the patient before making important decisions.

In these lines, the absence of a culture of teamwork and the lack of leadership, associated with time pressure, an excessive number of cases and the problems of attendance of professionals can lead to the suboptimal performance of LC-MDTs and a difficult decision-making process [6].

A representative attendance of all the specialties to the meetings is an important factor for the effective work of MDTs. Nowadays, the challenges associated with the location of the different specialists of a multidisciplinary model can be resolved by holding the meetings online or by telephone [18].

The fear of physicians to lose access to patients after referring them to another site for their MDT evaluation is another common limitation [18]. Likewise, skepticism about the beneficial effects of a multidisciplinary approach for patients at an advanced disease stage has also been reported [18].

Multidisciplinary cancer care is recognized in all clinical guidelines, and in both national and international strategic plans. However, the institutional support for the development of quality and excellence MDTs in our environment is unfortunately limited. Health authorities must favor their professionalization, support their organizational development and provide them with resources to develop their activity with the highest standards.

### Implications for patients

The number of patients discussed at a MDT meeting can be very different among hospitals. While high-volume hospitals offer at least 1 weekly meeting, smaller institutions may not have enough cases to hold a meeting on a weekly basis, and therefore patients have to wait longer for a care management plan. A similar problem arises for rare tumors, such as mesothelioma; the lack of similar cases in small institutions may lead to poor knowledge and scarce skills in their management, and therefore these patients need to be referred to regional specialists [19]. Patients in Europe are also subject to inequalities in the access to high-quality, both among and within countries [1].

The emergence of precision medicine and the higher survival of the population entail new challenges for physicians, who must face an increasingly complex scenario that requires a constant update in molecular biology and available treatments to maintain excellence in patient care. The lack of qualified professionals can not only limit the availability of the latest therapeutic advances, but also the access to current standards of care for LC patients. In these lines, access to an accredited molecular pathology laboratory should be guaranteed if it is not available in the hospital [1]. On top of that, patients without access to public healthcare systems or with domestic problems, general health impairment, comorbidities or toxicities may have extra difficulties in following the LC-MDT recommendations. These situations must be documented and analyzed when they occur [13]. At last, the limitations of a patient to be evaluated at a LC-MDT may affect access to clinical trials [8, 9]. A network of clinical trials at a national level would be a solution in this sense.

## Staff of LC-MDTs

**Fig. 1:**
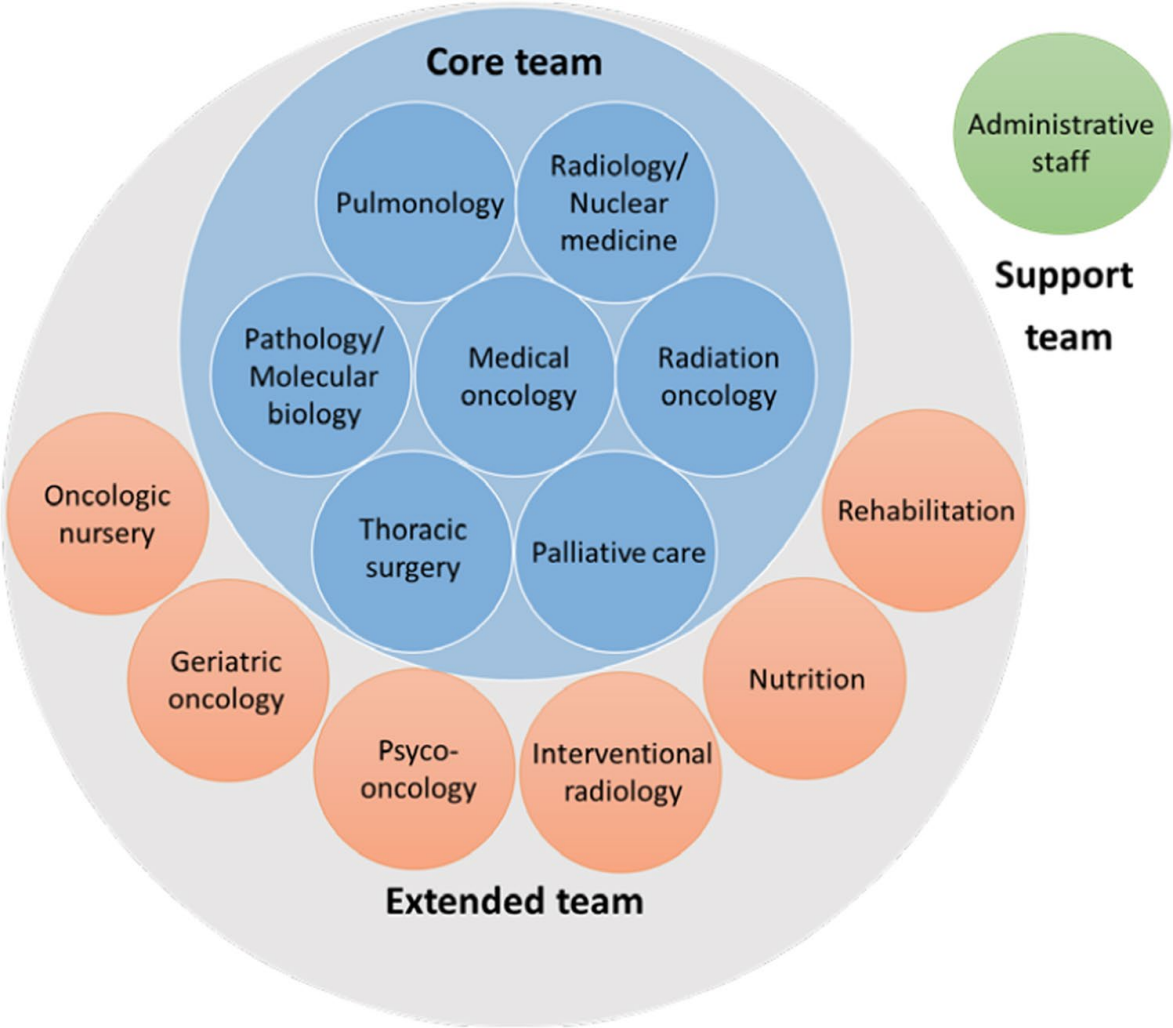
Core, extended and support team members of a lung cancer multidisciplinary team

MDTs are fundamentally constituted by a nucleus of specialists involved in the key steps of the assessment and management of patients with a suspected or confirmed diagnosis of LC (core members), and by a group of professionals from other areas that contribute throughout the process to increase the quality of the service provided (extended members) (Fig. 1) [1, 20].

### Role of core members

Core team encompasses pulmonologists, radiologists and nuclear medicine specialists, pathologists and molecular biologists, thoracic surgeons, medical oncologists, and radiation oncologists [1, 7, 10], who must be present in all MDT meetings [19], either in person or virtually.

- Pulmonologists usually manage chronic pulmonary conditions that confer an increased risk for the development of LC. They are the first specialists consulted in the presence of LC suspicion, and they are in charge of organizing diagnostic and staging procedures, such as endobronchial ultrasound-guided transbronchial needle aspiration (EBUS-TBNA); they assess lung function, and intervene in the treatment of respiratory comorbidities, in the management of pulmonary toxicities and in the performance of palliative maneuvers [1].
- Radiologists are in charge of performing clinical staging and radio-guided biopsies both at the first diagnosis and at progression of the disease or relapses. The role of radiologists is crucial in the assessment of the response to treatment and in the diagnosis of complications and toxicities. Activation of rapid diagnostic pathways after the iconographic finding of a suspicious lesion are also some of radiologists’ responsibilities [1, 21].
- Specialists in nuclear medicine are gaining an important role in LC-MDTs, as fluorine-18 fluorodeoxyglucose positron emission tomography/computed tomography (^18^F-FDG-PET/CT) has shown to be effective in selected indications, such as staging, guidance for targeting curative-intent radiotherapy or chemotherapy or restaging [1].
- Pathologists are responsible for the processing of samples, the histological confirmation of LC suspicion, and the performance of additional tests, such as specific immunohistochemistry. They decide the best sample and select the tumor area to perform molecular tests doing it in the shortest time to avoid delays in the start of treatments [13, 22].
- Molecular biologists are lately being incorporated in MDTs due to the growing use of targeted therapies, which have been associated with increased survival rates [23]. They are essential for the reading and interpretation of molecular tests, obtained from both tissue samples or liquid biopsies [1].
- Thoracic surgeons are involved in the diagnosis, staging, and curative and / or palliative treatment of LC [17]. They are the ones to evaluate resectability of LC lesions. Minimally invasive techniques are the current choice to reduce the morbidity associated to surgical procedures at early stages of LC [1].
- Medical oncologists play a role in the management of patients with LC, regardless of the stage [1]. They must ensure that patients are completely and accurately diagnosed and staged, which is essential for a subsequent correct therapeutic planning. The therapeutic approach chosen depends on the molecular subtype and the stage of LC, as well as on the functional status, comorbidities and preferences of patients. The knowledge and management of systemic toxicities and the facilitation of early access to clinical trials are other of their roles [24–26].
- Radiation oncologists must define and administer radiotherapy treatment with curative, palliative or prophylactic purposes. They also determine the dose-fractionation prescription and define the target volume and organs at risk [1].
- Palliative care specialists could be part of the core or the extended LC-MDT. Their participation in the MDTs has been associated with an improvement in the quality of the service provided, survival, quality of life, adherence, and patients’ and caregivers’ satisfaction, with a reduction in referral times and unnecessary visits to the hospital, and with a smooth transition between the services involved in the patient’s care [2, 13]. In particular, it has been demonstrated that early palliative care leads to significant improvements in survival, quality of life and mood of patients with advanced non-small cell lung cancer [27].

### Role of extended members

The incorporation of extended disciplines to MDTs provides precision and quality to the decisions made, adapting them to the circumstances and reality of patients.

- Nurses are a key figure in multidisciplinary assessment [7, 19, 28]. Psychosocial support, education of patients and caregivers, management of treatment-related toxicities and side effects, appointments coordination, and liaising between the patient and the medical team are some of the main duties of nursing staff [7].
- Specialists in geriatric oncology can facilitate global geriatric assessment of elderly patients, report on the frailty of the patient and an estimate their life expectancy, which may help prioritize the medical interventions proposed by MDTs [29].
- More than one-third of oncologic patients present with anxiety or depression. Psycho-oncologists may help address the psychological needs of oncologic patients and their environment, and promote effective communication between patients, families, and healthcare staff [1].
- Interventional radiology, rehabilitation and nutrition are other disciplines with a potential relevant role in the comprehensive approach to patients with LC, and consequently, with a relevant contribution to LC-MDTs [1].

To guarantee equity and quality of care in the event of lacking any of the crucial personal or technical resources, direct access to them needs to be provided by, for instance, sharing centralized services. These services cannot be implemented in all centers, because specific and expensive human and material infrastructure is required. Thus, telemedicine can be a useful tool to allow the connection within professionals from different centers, overcoming physical distance.

## Team skills and leadership

Technical and personal qualification play a fundamental role in the composition of the MDT [17]. Communication, leadership and relationships between members are key to ensuring efficient MDTs [19]. Interpersonal relationships among the MDT members are relevant and must be based on respect, communication and trust, to generate good teamwork, open mind and broadly-agreed decisions [14, 30].

Individual personalities can adversely affect the functioning of MDTs, in terms of holding reasonable discussions or make democratic decisions. Different opinions in what guidelines are useful for a certain case can exist, and therefore, good leadership will be essential for the functioning of the MDT and for achieving patient-centered and consistent decisions [19, 20]. The leader figure is crucial to guarantee an equitable participation of the different disciplines; the quality of decisions and their adherence to clinical guidelines and scientific evidence; the coordination of the actions to be carried out; the simplification of hospital bureaucracy and healthcare processes; the compliance with timings; the audit; and the motivation to improve the quality of healthcare. Leadership can have a rotating nature, which has been shown to contribute to enhancing teamwork and interpersonal relationship [6, 10, 20, 31]. Although requirements for leadership relate to coordination and management of hospital departments [19] and any MDT member may be the coordinator of the LC-MDT, clinical nurses are good candidates to take the leadership, with certain advantages such as greater performance and effectiveness of MDTs [32]. Furthermore, pulmonologists have also typically played the role of MDT leader to avoid treatment biases by other specialties [19].

In any cases, due to the constant incorporation of great advances in the diagnosis and treatment of LC (molecular biology, genetic counseling, bioinformatics, radiobiomics, or screening, among others), LC-MDTs must be dynamic decision-making bodies that gradually adapt to the emerging needs.

## Modalities of LC-MDTs

A huge variability of MDTs has been described in the literature in different countries, regions, or even inside the same healthcare areas [1, 7]. Two modalities have been defined according to the different levels of care of hospitals and the medical specialties available in the portfolio of services:

- Intra-hospital LC-MDTs, which are organized for those hospitals that do not have access to all the specialties of the core team.
- Reference LC-MDTs, which are organized in reference hospitals with all the specialties of the core team. Reference LC-MDTs usually receive cases from those hospitals without certain essential specialties in their LC-MDTs. All patients who require multidisciplinary approach should be evaluated in these committees, either in person or online [1, 33].

Two other modalities can be described, depending on the attendance of the MDT members:

- In person, with physical attendance of the essential members of the LC-MDT. The main advantage is the contact between professionals, which ease communication and shorten the time for decision-making.
- Online, which has been especially relevant during the COVID-19 pandemic, ensuring quality of care for patients with LC. Likewise, this modality is useful in hospitals with long distances to the reference center and allows the optimization of time and resources [7, 19, 20, 34]. Some advantages of online meetings are an increased comfort for attendees, a reduction in costs by eliminating travel time to a central location, and an easy incorporation into the schedules. Moreover, most specialists owe a computer with internet access and a telephone line at present, so requirements are mainly low [34]. Tools and facilities for good quality videoconferences are essential to allow the participation of the members who cannot attend the meeting in person or who work in other hospitals [35].

## Resources

### Physical space

The room where LC-MDT meetings are held should be in a quiet place and properly soundproofed to preserve confidentiality. Its size and distribution must be adequate to provide a seat to all the members, to see and hear each other, and to see the tests and diagnostic images of the presented cases [20].

### Technology and equipment

The rooms must be equipped, at least, with the following tools [20]:

- Equipment for projecting and view images of the complementary tests [20].
- Possibility to contact the computer service of the hospital in a timely fashion to resolve technical incidents that could affect the decision-making process [20].
- Access to the database where presented cases and the decisions made are recorded [20].
- Tools and facilities for good quality videoconferences with members who are off site, by which the reports and images of complementary tests can be shared and accessed [20].

Computer tools such as NAVIFY Tumor Board Solution (NTB) are useful, since they help organize the list of patients to present, and give access to clinical information and relevant complementary tests; they show a significant impact on the time for the preparation of MDTs, as well as in the homogenization of the format of all cases [36]. When these tools are not available, it is highly recommended to have a single model for electronic records of all the different hospitals that participate in the MDT meetings. Certain artificial intelligence systems have demonstrated to be useful for the decision-making in LC-MDTs [37, 38].

## Organization of LC-MDT meetings

### Before the meeting

Meetings must be held regularly. The cases presented should be included in the MDT list/agenda in advance, before an agreed deadline, although there should be flexibility for the addition of last-minute cases whenever it is justified. For the meeting call, communication systems that guarantee privacy of the information should be used. The cases presented need to be sent to the administrative staff, and they will be available to all members of the MDT. Meetings must be held during ordinary working hours, and this time consumption must be considered when clinical departments organize their activity [7, 20, 31].

Administrative staff is crucial for the effective and efficient functioning of the LC-MDTs, as well as for the coordination of procedures for adequate patient care [10]. The lack of administrative coordinators has been described as a major barrier for MDTs success [30], as otherwise, the team members have to take organizational roles, thus increasing their workload. Administrative staff can be responsible for the call, the preparation of the agenda and the writing of minutes of MDT meetings.

### During the meeting

Every physician presenting a case must attend the meeting or delegate to another member of the LC-MDT. Support of administrative staff is essential for the LC-MDT meeting; they can be in charge of completing the minutes, whose draft needs to be prepared before the meeting, and of recording the conclusions and decisions made during the session [20].

Time for holding the meetings must be sufficient to avoid discussing too many cases in short periods of time [19]. The time necessary for the discussion of complex cases must be always guaranteed. Depending on the MDT modality, either all cases or a selection of those more complex or that hardly fit in the protocols, must be discussed. In the latter case, those cases not discussed need to be registered anyways. An agreed minimum data set for all patients should be collected and summarized to be discussed during meetings; this should include diagnostic information, clinical information (comorbidities, psychosocial and palliative care needs) and patient history, as well as, when known, the patient’s opinions and preferences. LC-MDT meetings must run dynamically. For this purpose, all the information related to a specific clinical case must be available and easily accessible at the time of the meeting. For this purpose, a leader or a coordinator, who defends the needs of the committee, is essential to guarantee its operation, organization and execution.

The activity dedicated to the LC-MDT (either preparation of the cases or the attendance to the meeting itself) should be considered as a well-defined healthcare activity, which requires a regulated exclusive time. The implementation of some cultural and organizational changes led by the MDT and supported by the health administration is crucial for this purpose.

### After the meeting

The responsible physician must inform the patient of the therapeutic decision as soon as possible. If the LC-MDT has decided to carry out complementary tests, it should be managed effectively. The referral to specialists who will continue with the care process should also be effectively managed; this information can be communicated to LC-MDTs of other hospitals that may be involved in the care process. The decision must be recorded in a format accessible to all the professionals involved in the case itself [7, 19, 20]. When different appropriate decisions about treatment arise, these options should be presented to the patient in an unbiased manner.

### Registration of LC-MDT activity and legal implications

One of the aims of a LC-MDT must be to save steps in the healthcare process, as well as to simplify the hospital bureaucracy [31]. LC-MDT meetings are official sessions, and as such, minutes should be recorded, including the attendees and the clinical decisions that have been made. Ideally, these decisions should be incorporated in the patient’s medical records by computer support.

Institutions must take measures to support the LC-MDTs, as concerns regarding the medico-legal implications of the decisions made under their umbrella may arise.

## Presentation of cases

### Patient profiles

Each LC-MDT must establish which patients are candidates to be presented at meetings [20]. According to expert opinion, the different clinical scenarios for which a patient is susceptible to entering the list can be summarized into four large groups:

- Patients with new diagnosis of LC
  They are usually managed by the pulmonology team. When these patients are presented, diagnostic techniques such as CT, fiberoptic bronchoscopy (FOB) to obtain a cytological sample or bronchial biopsy, or transthoracic puncture have already been performed; staging by EBUS-TBNA, mediastinoscopy, or PET/CT is also necessary [1].
- Patients with recurrence or factors that imply changes in the therapeutic plan
  Sometimes, additional tests that may entail a change in the therapeutic plan have not been performed before the first presentation (stress test, perfusion scintigraphy, cranial CT, etc.), and may require a new LC-MDT evaluation. These cases are usually presented by the first diagnostic team (usually pulmonology) but also by other teams, such as thoracic surgery. On the other hand, patients who are already being managed by the medical or radiation oncology teams may undergo reevaluation at some point in the presence of certain findings (usually after chest CT scan). Afterwards, whether treatment needs to be changed has to be evaluated. Likewise, recently operated patients whose final anatomopathological diagnosis is decisive to conclude the treatment, to follow-up or to offer an adjuvant systemic treatment can also be presented to the LC-MDT by the pathology or the thoracic surgery teams.
- Patients who undergo rebiopsy
  The importance of rebiopsy is well known in lung cancer. The rebiopsy is used to complete the pathological diagnosis, molecular diagnosis or to study the acquired resistance to a certain treatment to redirect the best therapeutic approach. In those patients where treatment was selected according to tissue characteristics, and changes have been found, the treatment selection process should be performed again by the LC-MDT [39].
- Patients with treatable molecular targets
  The complexity of offering a precision oncologic treatment may lead to the creation of a molecular committee. Depending on the hospital, this can be integrated into the same MDT; carried out together with the molecular findings of other non-pulmonary tumors; or be exclusive for LC. Molecular tests to guide treatment decisions can be carried out within the same hospital, in a molecular pathology and / or biology department, or as an external service outside the hospital [40].
- Patients presented by external teams
  The emergency, the internal medicine or other teams can also present cases in MDTs meetings. Patients on the transplant list with a pulmonary nodule discovered; patients with pleural effusions with symptoms who require invasive diagnosis and treatment; patients with oncological diseases of other organs and with pulmonary nodules that require differential diagnosis and treatment, are some examples.

### Presentation and recording

To be presented to a LC-MDT, a patient must have a diagnosis of a mediastinal or pleural nodule, or a lung mass suspicious of malignancy by radiological image, although in some cases it will be a benign lesion.

A minimum clinical information needs to be collected in the presence of a suspicion, and a series of diagnostic tests such as imaging techniques (CT, PET/CT) should be started. These tests are crucial to assess the extent of the lung, mediastinal and hilar lymph nodular disease, and eventual metastases, as well as to identify the optimal site and method for sampling, establish the histopathological diagnosis (by FOB, EBUS, CT-guided puncture or biopsy, etc.), and define the tumor stage [17, 30].

It is recommended that physicians who are not members of the LC-MDT present their cases when they doubt on how to manage a certain patient. Consultation to the LC-MDT members on how to proceed for the presentation can be helpful, as they may suggest the petition for a diagnostic test before the presentation.

**Fig. 2:**
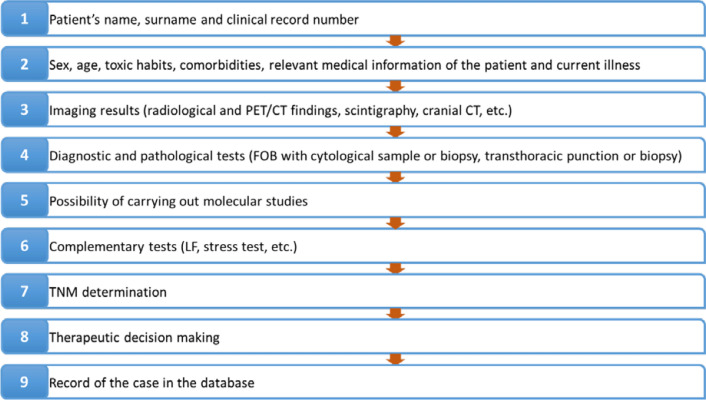
Ideal sequential order for the presentation and discussion of cases in lung cancer multidisciplinary team meetings. *CT* computed tomography, *FOB* fiberoptic bronchoscopy, *LF* lung function, *PET/CT* positron emission tomography CT, *TNM* tumor-node-metastasis staging

The authors suggest that the whole information of a case and the latter derived actions take place in a sequential order that has been summarized in Fig. 2.

In the case of patients who have been discussed in previous meetings, it is recommended to first offer a brief summary of the medical history and findings, subsequently provide the new information, and finally reach a consensus on the best treatment for the patient.

The records of the cases presented in LC-MDTs are valuable instruments for the control of the disease. They are essential for the evaluation of the diagnosis and treatment processes. Data such as age, sex, smoking habit, tumor type, stage, or therapeutic decisions must be recorded. This information can be useful for the assessment of quality indicators, for determining epidemiological data in our population, or for evaluating and controlling the impact of cancer in our community. Registration should be carried out by an expert member of the LC-MDT who is present during the meeting. Case managers are appropriate people to cover this role [20].

Given that these records are a source of information about every patient, their link to other databases such as the Spanish Registry of Clinical Trials or the Spanish Agency for Medicines and Health Products would be useful to increase the possibility of enrolling on a clinical trial at advanced stages of the disease.

## Excellence requirements and quality indicators

Quality indicators are defined as measurable elements of practice performance for which there is evidence or consensus that they can assess the quality of the care provided and the effect of certain changes on it [41, 42]. Many quality indicators specific for LC have been described to cover all the dimensions of care (from the healthcare professional to the patients’ perspective), and to achieve objectification of all processes [30, 43].

Following the characteristics of a LC-MDT described in this review, authors have compiled a list of excellence criteria that may help built new LC-MDT or transform those existing, following certain standards of quality; some of them are related to general aspects of LC-MDT, such as composition, organization and necessary resources (Table 1), while others are specific for each core specialty (Table 2). Moreover, the use of some quality indicators has been suggested to analyze the performance of LC-MDT and implement changes, if necessary (Table 3).

**Table 1:** Excellence requirements for LC-MDTs

Area	Excellence requirement
Staff	Members of LC-MDTs must belong to different disciplines and have experience in their fields
	Members of LC-MDTs must show respect for the patients and their colleagues, and favor an environment of fluid communication
	All the disciplines involved in LC-MDTs must be accessible for queries that may arise during the evolution of the disease, and show collaboration to network with other hospitals and specialists
	Members of LC-MDTs must have an accredited academic training that demonstrates their technical and professional skills
	There must be a leader who coordinates and channels the different opinions presented at LC-MDTs meetings [19, 20]
Resources	The room where LC-MDT meetings are held must be in a quiet place and properly soundproof to preserve confidentiality. Its size and distribution must be adequate to provide a seat to all the members, to see and hear each other, and to see the tests and diagnostic images of the presented cases [20]
	There must be sufficient technical resources for the performance of care, in terms of equipment, staff, computer resources, or physical space, for the proper functioning of every specialty
	LC-MDTs must count on administrative support [7]
	The format of presentation of the cases must be standardized, for what support computer tools may be used; it is highly recommended to use the same model of electronic record among the different hospitals that participate in the same LC-MDTs [7]
	Cases must be included in an agenda in advance, before an agreed deadline, with flexibility for the addition of justified last-minute cases. Communication systems that guarantee the privacy of the information must be used [20]
	Due to the increase of online LC-MDT meetings scheduled, tools and facilities for good quality videoconferences must be provided, by which reports and images of complementary tests can be shared and accessed [20]
Organization	Standard operating procedures must be written and periodically updated
	LC-MDT meetings must be held regularly, at a previously agreed time [7]
	Decisions made at LC-MDT must be recorded in a format accessible to all the professionals involved in the care process [7, 19, 20]
	Meetings must be held during ordinary working hours, and this time consumption must be considered in the organization of clinical departments [20]
	Patients must be informed of each step within the multidisciplinary process [1]
	LC-MDTs must meet at least once a year to review the activity of the previous period and audit the results, to carry out changes in protocols and procedures and improve the performance of the unit / center, when needed [1]
	It is recommended to hold regular meetings to update and analyze the LC-MTD objectives, and to discuss management issues
	All LC-MDT decisions must be documented in an understandable way and be part of the patient’s records [1]

*LC-MDT* Lung cancer multidisciplinary team

**Table 2:** Excellence requirements for LC-MDTs core team members

Area	Excellence requirement
Pulmonology	Pulmonologists must be able to interpret imaging studies and have experience in diagnostic and palliative bronchoscopic techniques [1]
	Those pulmonologists administering medical therapy must meet the requirements of medical oncologists [1]
Radiology/nuclear medicine	Radiologists must be familiar with: management of pulmonary nodules; strength and limitations of bronchoscopic interventions; image guided biopsies and radiological treatment options; treatment responses to radiotherapy, chemotherapy, targeted therapy and immunotherapy, and their adverse events; and surgical procedures [1]
	Radiologists must have knowledge about: patterns of lymphatic and hematogenous spread of LC; TNM staging system; and when to refer to nuclear medicine for PET/CT [1]
	Nuclear medicine physicians must have expertise in PET/CT [1]
	Nuclear medicine departments must be able to perform verification protocols and to react accordingly [1]
Pathology/molecular biology	Pathologists must count on diagnosis of the cases that are to be presented at each MDT meeting
	Pathologists must know the material received for the cases to be presented at each MDT meeting, to guide MDT future steps in matters of new diagnostic tests or request of new sample, in case of scarce material
	Pathologists must be familiar with pathological TNM for the diagnosis of cases undergoing surgery and be aware of the latest developments in terms of diagnosis after neoadjuvant treatment
	Molecular biologists/ pathologists must know which patients should undergo molecular characterization, which genes should be tested with priority, and whether a rebiopsy is needed [13, 44–46]
Thoracic surgery	Thoracic surgeons must know the surgical indications for LC, as well as the different diagnostic and therapeutic approaches [1]
	Thoracic surgeons must be able to identify and, when possible, to resolve potential complications of the procedure performed, during both the surgery or the postoperative period [1]
Medical oncology	Medical oncologists must individualize the treatment to be the least toxic, the safest and the most cost-effective, based on the overall characteristics of the patient [24]
	Medical oncologists play a fundamental role in helping to select the appropriate diagnostic techniques for optimal characterization of tumors, to choose the best treatment based on the patients’ specific anticancer targets [26]
	Medical oncologists must be responsible for updating and training the rest of the committee in the availability of new drugs and their indications, as well as for facilitating early access to clinical trials that may represent an opportunity for patients [25]
Radiation oncology	Radiation oncologists must know the indications for radiotherapy (whether curative or palliative); the most appropriate techniques to perform it; and the criteria for the selection of patients subsidiary of radiotherapy, alone or associated with other therapies [1]
	Radiation oncologists must be aware of the benefits associated with radiotherapy treatment (survival, local control), possible adverse effects and impact on quality of life [1]
Palliative care	Palliative care must provide relief from pain, stress and other symptoms to improve the quality of life for the patient and their families [47]
	The palliative care team must be introduced early in the treatment of disease to improve quality of life and even overall survival [47]

*LC* Lung cancer, *LC-MDT* Lung cancer multidisciplinary team, *PET/CT* Positron emission tomography-computed tomography, *TNM* Tumor-node-metastasis staging

**Table 3:** Quality indicators for LC-MDTs

Quality indicator	Measure	Proposed standard	Justification
General			
Availability of a LC-MDT	Does the LC-MDT exist at the site?	Yes [1]	LC-MDTs are fundamental structures for the diagnostic and therapeutic approach of patients with LC
Normalized procedures of structure, organization and functioning	Does the LC-MDT count on normalized procedures of structure, organization and functioning?	Yes [1]	LC-MDTs must be multidisciplinary and organized so that the activity is based on professional knowledge and skills and on agreed decision-making
Periodic report of the LC-MDT activity	Does the LC-MDT prepare an annual activity report?	Yes [1]	Continuous evaluation, activity monitoring and improvement are necessary
Independent evaluation of the LC-MDT activity	Is there an external audit at least every 3 years?	Yes	A positive external evaluation guarantees the quality of the LC-MDT performance
Quick access to relevant clinical information	Does the LC-MDT have a system for accessing clinical data?	Yes	LC-MDTs must have electronic access to any type of relevant information for decision-making, as well as technical support to be able to present it appropriately
Record of clinical decisions	Does the LC-MDT have a system for recording activity?	Yes	LC-MDTs must have an agenda or electronic folder where decisions are recorded, thus ensuring the traceability of clinical decisions The treatment plan must be available to all members of the LC-MDT and must be included in the electronic records
Computer management tools	Does the LC-MDT have an electronic platform for managing clinical cases?	Yes	LC-MDT functioning can be optimized through computer applications to manage information for decision-making, as well as with traceability and automatic preparation of minutes of meetings
Agenda organization	Is the participation of members on the LC-MDT included in their work agenda?	Yes	LC-MDT activity must be considered as a healthcare activity by organizations and requires exclusive dedication time
Continuous update of clinical protocols	Does the LC-MDT update annually the clinical protocols?	Yes	It is necessary to incorporate scientific findings into clinical practice
Involvement in multidisciplinary research	Is the LC-MDT involved in research projects?	Yes	Multidisciplinary research favors communication between specialties and can positively impact in the care of LC patients
Educational retrospective review sessions	Does the LC-MDT hold annual review sessions?	Yes	Retrospective review of cases has an educational aim and contributes to continuous improvement of decision-making
Participation in clinical trials	Do patients evaluated by the LC-MDT have options to participate in a clinical trial?	Yes	Clinical trials may represent an opportunity for patients with cancer. LC-MDT members must facilitat early access to them
Clinical implementation of LC-MDT decisions	N presented to the LC-MDT in which the decision agrees with the treatment administered/N presented to the LC-MDT × 100	> 90%	LC-MDT decisions are evidence-based and supported by guidelines and multidisciplinary agreed protocols. In the face of a deviation from the initial recommendation, cases must be resubmitted and changes must be justify and recorded
Efficiency of the LC-MDT	N with LC included in more than one session of the LC-MDT*/N with LC included in the LC-MDT × 100	< 5%	The repeated presentation of cases without the necessary tests for decision-making is one of the main inefficiency problems of LC-MDTs
Multidisciplinary evaluation of patients with a new diagnosis	N with a new diagnosis of LC evaluated in the LC-MDT/N with a new diagnosis of LC × 100	> 90% [48]	Decision-making must be based on the exchange of knowledge and experience among the different specialties
Multidisciplinary evaluation of patients with recurrence	N with recurrence evaluated in the LC-MDT/N with recurrence × 100	> 90% [48]	Decision-making must be based on the exchange of knowledge and experience among the different specialties
Multidisciplinary evaluation of patients after radical surgery	N after radical surgery evaluated in a tumor committee/N after radical surgery × 100	> 90% [48]	Decision-making must be based on the exchange of knowledge and experience among the different specialties
PET staging in patients subsidiary for potentially curative treatment	N presented with curative intent in the LC-MDT with PET/N presented with curative intent in the LC-MDT × 100	100% [49]	PET is crucial for the proper staging of LC

*LC* Lung cancer, *LC-MDT* Lung cancer multidisciplinary team, *N* Number of patients, *PET* Positron emission tomography

*Excluding cases revaluated after surgery or recurrences

## Conclusions

To summarize, MDTs are necessary to optimize health outcomes and improve care of patients with LC. Time, leadership, teamwork and certain resources are crucial for preparing and holding LC-MDT meetings. The fulfillment of the exposed excellence requirements and the implementation of the proposed quality indicators may help to achieve the highest quality performance of current LC-MDTs.

## Abbreviations

CT: Computed tomography
EBUS: Endobronchial ultrasound
FOB: Fiberoptic bronchoscopy
^18^F-FDG-PET/CT: Fluorine-18 fluorodeoxyglucose positron emission tomography/computed tomography
LC: Lung cancer
LF: Lung function
MDT: Multidisciplinary team
NSCLC: Non-small cell lung cancer
NTB: NAVIFY Tumor Board Solution
PET/CT: Positron emission tomography
TBNA: Transbronchial needle aspiration
TNM: Tumor-node-metastasis staging
